# Effect of Cognitive Behavioural Stress Management on Return-to-Work Amongst Sick-Listed Employees

**DOI:** 10.1007/s10926-025-10306-2

**Published:** 2025-06-25

**Authors:** Charlotte Brøgger Bond, Morten Vejs Willert, Daniel Navy Ditlevsen, Louise Fleng Sandal, Lars Brandt

**Affiliations:** 1 Department of Sports Science and Clinical Biomechanics, University of Southern Denmark, Odense, Denmark; 2 Department of Occupational Medicine, Danish Ramazzini Center, Aarhus University Hospital, Aarhus, Denmark; 3 Regional Hospital, Gødstrup, Denmark; 4 Department of Occupational and Environmental Medicine, Odense University Hospital, Odense, Denmark; 5 Department of Clinical Research, University of Southern Denmark, Odense, Denmark

**Keywords:** CBT, RTW, Sick leave, Stress management, Job

## Abstract

**Background:**

The literature provides contradictory information on the effect of cognitive behavioural therapy (CBT) interventions on return to work (RTW) for patients on sick leave due to work-related stress.

**Aim:**

We compared the cumulative number of sick leave weeks between a cohort of patients who received a CBT-based stress management intervention in the period 2011–2018 (*N* = 331) and a cohort of patients from 2010 to 2011 (*N* = 221) who did not receive the intervention. We also compared time until sustainable RTW (3 consecutive months of no sick registrations) between the cohorts.

**Methods:**

Registry data on sickness absence were obtained from the national DREAM register (Danish Public Transfer Payments Database). The cumulative time of registered sick leave in DREAM until first sustainable period of RTW was visualized using Kaplan–Meier plots. A Cox proportional hazard model was used to compare the effectiveness of the intervention relative to the comparison cohort and was reported as hazard rate ratio.

**Results:**

The intervention cohort’s cumulated number of weeks in DREAM across the total period from 0 to 36 months (median 29 range 26–32) was lower than that of the comparison cohort (median 40 range 34–52), (*P* = 0.005), corresponding to a 14% (95% CI 0.04–0.24) reduction. For RTW, a hazard ratio rate of 1.25 (95% CI 1.02–1.54) favouring the intervention group was found (*P* = 0.034).

**Conclusion:**

The CBT stress management intervention significantly reduced the amount of sick leave and reduced the time until sustainable RTW for the intervention cohort compared to the comparison cohort.

**Supplementary Information:**

The online version contains supplementary material available at 10.1007/s10926-025-10306-2.

## Introduction

Common mental disorders such as depression, anxiety, and adjustment disorder are a leading cause of sick leave in the Western World [1–3]. Specifically, work-related stress (e.g., adjustment disorder, burnout, and overexertion) exerts heavy economic burdens due to sick leave across countries [4]. Work-related stress is defined by World Health Organization (WHO) as a potential reaction when people experience work demands and pressures that exceed their coping capacity [5]. Work-related stress is associated with increased risk of cardiovascular [6, 7], musculoskeletal diseases [8], and mental disorders (depression and anxiety) [9, 10]. In addition to the personal suffering, it is costly for society in terms of sick leave benefits, treatment costs, and lost productivity [11]. Whilst preventing work-related stress remains a key public health priority, it is also important to provide effective and relevant stress management interventions to support recovery and facilitate return to work (RTW) for those affected.

Cognitive behavioural therapy (CBT) interventions for work-related stress are effective on lowering self-reported stress, anxiety, and depression [12]. However, the effect of CBT interventions on duration of sick leave and return to work (RTW) is less clear [2].

Whilst a recent systematic review and meta-analysis found CBT effective in reducing the length of sick leave and facilitating RTW, great heterogeneity was reported regarding various settings (e.g. studies included several nationalities with different healthcare systems and social policies), choice of outcomes, scales, and endpoints (some of the RCTs measured at the end of the intervention whilst others were measured a few months after the intervention had ended) [13]. Another systematic review with a narrative synthesis reported that work-focused CBT and problem-solving skills facilitate a faster RTW amongst employees on sick leave for common mental disorders (depression, anxiety, and stress) compared to treatment as usual. However, no consistent effect on full RTW was found [14]. The heterogeneity in the literature and inconsistency in results points to a need for more evidence with well-documented clinical populations, descriptions of settings and outcome measures to assess the generalizability of these findings to other contexts. Additionally, there is a scarcity of replication studies where the same intervention is evaluated in a different setting.

In Denmark, the public social security system ensures that employers can receive economic compensation for long-term sick-listed employees to lessen the burden of productivity loss and costs of temp hires. The first 30 days of long-term sick leave must be covered by the workplace, before compensation claims can be made. After 22 weeks of compensation, the municipality must evaluate if the compensation can continue, which is possible in most cases. Work-related stress is a main cause of long-term sick leave in Denmark [15]. Up to one-third of Danes participating in a national survey in 2021 reported a high score (> 18) on the Perceived Stress Scale [16, 17]. Another survey of the Danish working population reported that 14% always or often felt stressed with 61% reporting work as the main stressor [18].

In a recent register-based study of patients at the Danish Occupational Medicine Departments 85% of those diagnosed during 2010–2013 with stress disorder (diagnoses F43.2/8/9 or Z56.3 (ICD-10)) were working 2–5 years prior to assessment. At the time of assessment, most had entered long-term sick leave and only 30% were working. During the first year of follow-up approximately 60% had RTW, with no further increase up to 5 years later [19].

This study will utilize Danish registry information to investigate RTW for a group of patients who participated in a CBT-based stress management intervention at a Danish Occupational Medicine Department in 2011–2018 against a control group of patients who, in the years prior to introducing the CBT intervention, were given only an assessment, including guidance on RTW, but no CBT intervention. Willert et al. [20] evaluated the effect of the same CBT stress management intervention against a wait-list control in 2006–2008 in a randomized controlled trial (RCT). They found a tendency towards fewer weeks of sick leave and faster RTW rate for the intervention compared to the wait-list control, although statistically insignificant. Based on the findings of Willert et al. [20] we hypothesize fewer weeks of cumulated sick leave and a faster RTW rate for patients receiving the intervention as compared to the control group. Our objectives are as follows:

1. To examine if the CBT intervention lowers the proportion of sick leave in a group of patients who received the intervention in the period 2011–2018 compared to a group of patients from 2011 to 2012 who did not receive the intervention.
2. To examine if the intervention results in faster sustainable RTW for those on sick leave at the time of assessment in the intervention group compared to the control group.

## Methods

### Study Design

The study is a quasi-experimental cohort study with an intervention group (I-group) and a comparison group (C-group) of patients diagnosed with work-related stress (diagnoses F43.2/8/9 or Z56.3 (ICD-10)). The outcomes were cumulated number of weeks with sick leave registrations in time windows and time until sustainable RTW, the latter defined as returning to work or education for a minimum of 3 months without any sick leave compensation. The study is reported following the STROBE guideline [21].

### Setting

The Department of Occupational and Environmental Medicine in Odense, Denmark (DOEM) is based within the Danish health care system at a secondary level/hospital level, and patients are referred to the clinic from their general practitioner (GP). DOEM has offered CBT-based stress management intervention for work-related stress since 2011. Patients referred to DOEM are offered the intervention free of charge and attendance agreed upon with the employer as part of the plan for RTW.

### Participants

Patients were referred to DOEM because their GP suspected work-related stress.

Patients in the I group were recruited from 2011 to 2018. A psychologist at DOEM performed the assessment interview to evaluate if patients were eligible for treatment at DOEM. Patients meeting the following criteria were offered the intervention: (i) exposure to stressful conditions at work (e.g. excessive workload, conflicting demands), (ii) working or on temporary sick leave, (iii) exhibiting severe signs of work-related stress that can be attributed to work-related exposures, (iv) physical, psychological and behavioural stress symptoms lasting > 4 weeks, and (v) if on sick leave: planned RTW during the intervention. Exclusion criteria were (i) stress primarily caused by bullying and/or harassment, (ii) long-term sick leave > 26 weeks up to referral, (iii) severe non-work stressors or life events, (iv) severe psychiatric conditions requiring treatment, (v) current abuse of alcohol and/or psychoactive stimulants.

Patients in the C-group were systematically selected by a trained psychologist from DOEM using a screening tool that covered the intervention’s inclusion and exclusion criteria. The psychologist reviewed patient records from 2011 to 2012 (i.e. time period before offering the CBT intervention) and selected patients who would have been eligible to receive the intervention if the intervention had been a permanent offer at the time.

### Intervention

The CBT stress management intervention was group based (eight to nine patients per group) and consisted of nine sessions delivered over the course of 3 months. The content, exercises, and outline of the intervention were consistent throughout the recruitment period from 2011 to 2018, and minor updates to the visual material of the intervention were implemented in august 2018. Experienced psychologists led the group sessions following a treatment manual and received training by a senior colleague prior to leading group sessions [22, 23] (Supplementary file).

At session one, eight and nine patients were asked to complete questionnaires. We excluded patients who had not completed at least two out of the three questionnaires, using questionnaire completion as a proxy for attendance, as participants would have needed to be physically present at the sessions in order to complete them. Patients receiving the interventions (the I group) may have waited up to 6 months to start their intervention, as the intervention was conducted in groups and only started as when the total group number was reached.

### Data Sources

This study combined data from a patient cohort and a national register using a personal identification number (CPR) [24]. Data on participant and job characteristics were obtained from the Danish Occupational Medicine Cohort [25], which includes information from a number of national registers as well as the Danish Occupational Cohort (DOC*X) [26] on patients seen at the Danish Occupational Health Departments from 2000 to 2018. Data on sick leave compensation were obtained from the DREAM register (the Danish Register for Evaluation of Marginalization).

The DREAM register contains weekly registrations of receiving any public transfer payment, arranged as week-by-week panel data. Registrations of part- or full-time sick leave compensation begins after 30 consecutive days of sick leave and registrations terminate after a full week of no public transfer payments. The DREAM register currently contains 137 codes for types of public transfer payments, ranging, e.g. from sick leave benefits to education grants, retirement, and parental leave. For our analysis DREAM codes were divided into the following categories: (1) Working; (2) Education; (3) Temporary benefits; (4) Permanent benefits; (5) Retired; (6) Emigration; (7) Deceased; and (8) Maternity/parental leave. We subsequently categorized the outcomes into three groups: positive outcome (Categories 1 & 2), representing patients with an active work or educational status and minimal stress; negative outcome (Categories 3 & 4), indicating patients who would require temporary or permanent support from the system, suggesting continued stress, and neutral outcome (Categories 5–8), encompassing patients who were either retired, emigrated, or on maternity/parental leave. The neutral outcomes were censored from the analyses. Using the DREAM registry we were provided with panel data with a week time resolution for individual patients of their flow in and out of sick leave periods. The start date for the analyses was the assessment date for both groups. Patients in the I-group who had more than 6-month time difference between their assessment date and the actual start date for the intervention were excluded.

### Statistical Analysis

All data were obtained and analysed on a secure server in Statistics Denmark. The statistical analyses were performed using STATA (STATA, version 18, College Station, TX, USA).

Baseline characteristics of the two groups were compared using Chi^2^ test for categorical variables and Student’s *t* test for continuous variables. Any baseline characteristics that differed between the two groups were adjusted for in the time-to-event analysis.

To investigate the cumulated number of sick leave weeks we visualized transitions in and out of sick leave using sequence index plots. These allowed us to establish whether the I group and C group differed in patterns of sick leave. We then counted the cumulated number of sick leave weeks for each group in the time windows: 0–6 months, 6–12 months, 12–24 months, 24–36 months, and the full range of 0–36 months. Histograms of cumulated sick leave weeks in time windows showed, as expected, that the data were not normally distributed, therefore both the mean and median with corresponding 95% confidence intervals (CI) were reported. A Mann–Whitney *U* test investigate the difference between groups. As this is a quasi-experimental design, the number of patients is not balanced across groups. Consequently, the cumulated number of weeks with registrations in DREAM was calculated per 100 patients to allow for a more meaningful comparison between the groups. Additionally, a Somer’s D calculation estimated the percentage difference in cumulated weeks of sick leave between two randomly chosen individuals from each group in each time window and totally across 0–36 months.

To investigate difference in rate of sustainable RTW, we performed a time-to-event analysis. We defined three consecutive months of positive outcomes as sustainable RTW, in accordance with the definition in a recent systematic review [27]. Only patients in the I and C groups who were on sick leave at the time of their assessment were included in this analysis (I-group = 265, C-group = 181). The rate of RTW was measured as the cumulated number of weeks of registered sick leave in DREAM until first sustainable period of RTW and visualized using a Kaplan–Meier plot. A Cox proportional hazard model compared the return rate of the I-group compared to the C-group reported as hazard rate ratio (HRR). Model assumptions were investigated using a log–log plot and testing the proportional hazards assumption. We performed two sensitivity analyses to investigate the robustness of the findings: (a) a model reported as HRR adjusted for any baseline differences between the groups to address the non-randomized nature of the study design and (b) as the recruitment period to the I-group is 8 years as compared to only 1 year in the C-group, we investigated if the HRR differed over time by comparing I-group patients recruited from 2011 to 2014 and 2015 to 2018, respectively, against the C-group.

### Ethics

Project approval was obtained from Odense University Hospital according to the Data Protection Regulation. The study does not fall within the scope of the Medical Research Involving Human Subject Act (§14) and is therefore considered exempt from ethical approval by The Regional Committees on Health Research Ethics for Southern Denmark. The project is registered in ClinicalTrials.gov, number NCT05791461.

## Results

### Baseline Characteristics

Baseline characteristics are reported in Table 1. Differences were seen for level of further education, visits to psychologist during assessment year and occupational groups. The I-group had higher educational levels, with more patients in the category Managers and Professionals (includes occupations with a high level of education, such as engineer or nurse), whereas the C-group had more patients in the Services and Sales category (includes occupations with a lower level of education, such as eldercare workers and police officers).

**Table 1:** Baseline comparison

	Intervention group (*N* = 331)				Control group (*N* = 221)				*P* value
	*N*	%	Mean	Range	*N*	%	Mean	Range	
Age at assessment			45.23	7.80			44.37	9.15	0.23
Sex									0.051
Male	53	16.2			53	24.0			
Female	274	83.8			168	76.0			
Marital status									0.58
Married	200	60.4			125	56.6			
Single/widowed	85	25.7			59	26.7			
Divorced	46	13.9			37	16.7			
Level of further education^a^									0.003
Long	37	11			11	5.0			
Medium	179	54.6			103	46.8			
Short	89	27.1			79	35.9			
None	24	7.3			27	12.3			
GP visits in assessment year									0.54
0	23	6.9			18	8.1			
1–5	152	45.9			88	39.8			
6–10	94	28.4			67	30.3			
> 10	62	18.7			48	21.7			
Psychologist visits in assessment year^b^									0.010
None	307	92.7			216	97.7			
One or more	24	7.3			5	2.3			
On sick leave at assessment									0.53
No	67	20.2			40	18.1			
Yes	264	79.8			181	81.9			
Occupational groups									
(ISCO-88)^c^									< 0.001
Managers/professionals	108	40.0			54	24.4			
Technicians/associate professionals	100	37.0			80	36.2			
Clerical support workers	21	7.8			12	5.4			
Services and sales workers	28	10.4			55	24.9			
Manual workers and armed forces	13	4.8			20	9.0			
Comorbid somatic disease (from 1995 to date of assessment)^d^									
No	310	93.7			203	91.9			0.42
Yes	21	6.3			18	8.1			

^a^Level of further education: None = no further education after 9th grade or high school (12th grade), Short = 3 years after high school (e.g. eldercare workers), Medium = 4 years after high school (e.g. nurses), and Long = 5 years after high school (e.g. medical doctors)

^b^Psychologist visits in assessment year signifies all visits to a psychologist in the assessment year with a subsidized referral from the Danish health care system

^c^The occupational groups (ISCO-88) originally contain nine categories, as Statistics Denmark restricts reporting on groups fewer than five observations, the variable was collapsed into five groups. The variable had 61 missing for the intervention group. ^d^The variable comorbid somatic disease indicates whether a patient was diagnosed with a somatic disease by a physician from 1995 until their assessment

### Cumulative Number of Sick Leave Weeks

The cumulative number of weeks with registered sick leave is shown in Table 2 for each group. From 0 to 6 months, no statistically significant difference between the groups was observed. In all other time windows, however, a statistically significant lower amount of sick leave weeks was observed for the I-group. Across the total period from 0 to 36 months, the median number of sick leave weeks was 29 for the I-group as compared to 40 for the C-group. Totally, a difference of 1552 weeks of sick leave was found for the I-group compared to the C-group for every 100 patients. Consequently, being offered the CBT stress management intervention resulted in statistically significant fewer weeks of cumulated sick leave as compared to the C-group across the 3-year follow-up period and all the a priori specified time periods except the first 6 months.

**Table 2:** Register-based record of absenteeism from work, cumulative number of weeks registered as full-time or part-time sick leave in the DREAM register database, within time periods of 6- and 12-months intervals.

	Intervention group, *n* = 331		Control group, *n* = 221		*P*-value
	Weeks	95% CI	Weeks	95% CI	
Weeks in DREAM, 0–6 months					
Median	23	20–24	20	16–24	0.32
Mean	18	17–19	17	16–18	
Weeks pr. 100 patients	1813		1707		
Weeks in DREAM, 6–12 months					
Median	1	0–4	8	3–12	0.0024
Mean	8	7–9	11	10–13	
Weeks pr. 100 patients	807		1120		
Weeks in DREAM, 12–24 months					
Median	0	0–0	2	0–9	0.0002
Mean	9	8–11	18	15–21	
Weeks pr. 100 patients	1084		1792		
Weeks in DREAM, 24–36 months					
Median	0	0–0	0	0–6	0.0013
Mean	9	8–11	16	13–18	
Group total	936		1573		
Weeks in DREAM, 0–36 months					
Median	29	26–32	40	34–52	0.005
Mean	46	42–51	62	55–69	
Weeks pr. 100 patients	4640		6192		

*P*-values are from Mann–Whitney *U*-test statistics

95% CI = 95% confidence intervals.

Somer’s D was used to calculate mean difference in per cent between a random participant from each group. The results showed 5% (95% CI − 0.05–0.14) reduction in sick leave for the C-group in the first 6 months. In all other time periods statistically significant reductions in sick leave weeks were reported for the I-group. Across all 36 months of follow-up, the I-group had a 14% (95% CI 0.04–0.24) reduction in weeks of sick leave compared to the C-group.

### Rate of RTW

**Fig. 1:**
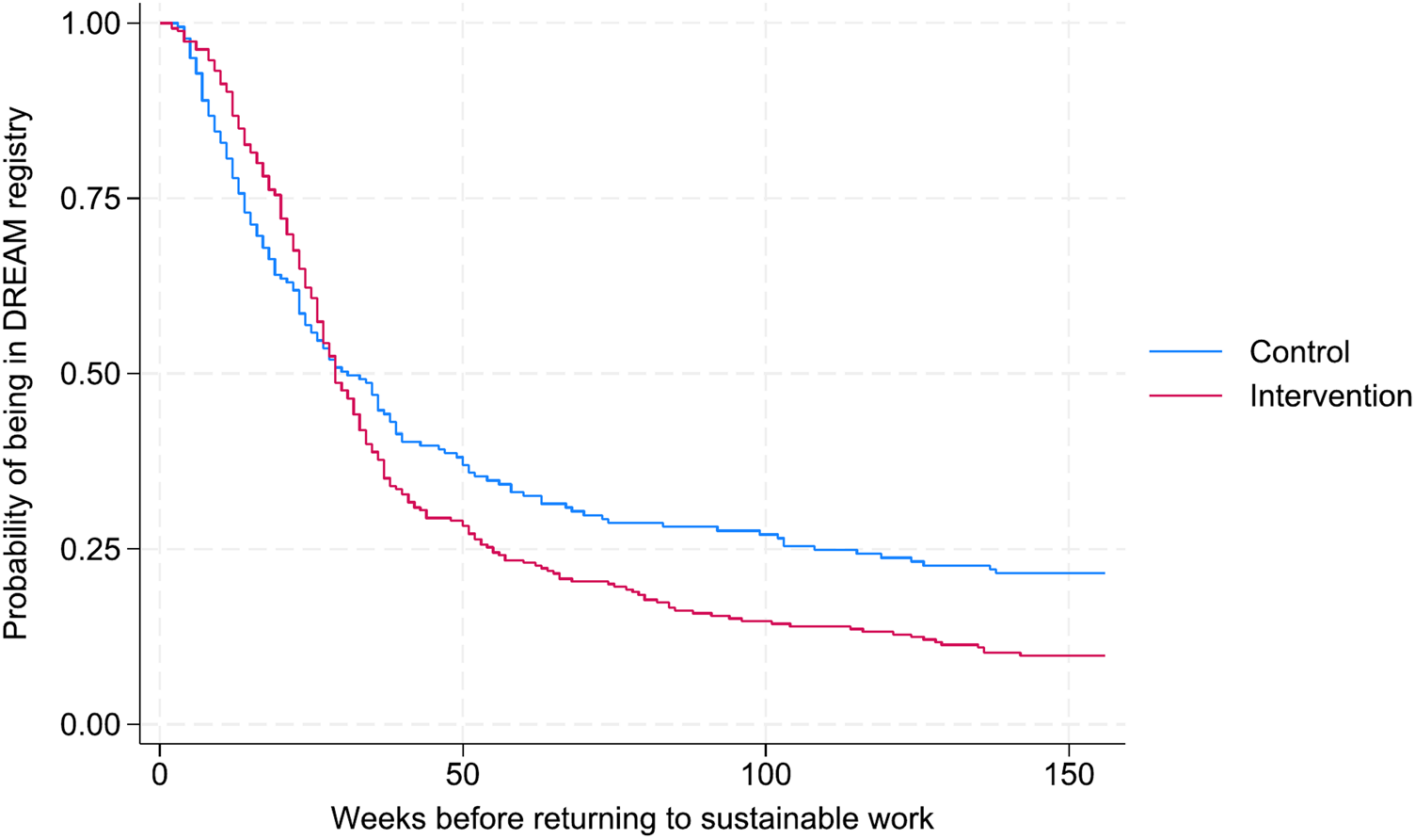
Kaplan–Meier plot of the rate of sustainable return to work, represented by the cumulative probability of being on full-time or part-time health benefits or unemployment. The x-axis increment is weeks. Sustainable return to work is defined as three consecutive months with a positive affiliation to the labour market

The rates of sustainable RTW for the patients who were on sick leave at assessment time (I-group: N = 265, C-group: N = 181) are shown in Fig. 1. The 25 percentiles of RTW were 20 weeks for the I-group and 14 weeks for the C-group. Median RTW was 29 weeks for the I-group and 31 weeks for the C-group. The 75 percentiles of RTW were 55 weeks for the I-group and 108 weeks for the C-group. A HRR for RTW of 1.25 (95% CI 1.02–1.54) between the two groups was found in favour of the I-group (p = 0.034). In our first sensitivity analysis, we adjusted for visits to psychologist and level of further education, the HRR was not affected (1.26 (95% CI 1.01–1.56). In our second sensitivity analysis, we limited the years covered in the I-group to 2011–2014 which did not affect the HRR (1.25 (95% CI 1.00–1.56)). We also limited the I-group to cover only years after 2014 and found that this did not affect the HRR (1.22 (95% CI 0.89–1.66)).

## Discussion

### Summary of Main Findings

This study found that the intervention reduced the total amount of time patients spent on sick leave and that patients who received the intervention had a faster sustainable RTW during a 3-year follow-up.

Significant fewer weeks of sick leave were observed for the I-group across all time windows except in the first 6 months. In total, a reduction of 1552 weeks was observed for the I-group when compared to the C-group pr 100 patients. At week 29, the RTW rate accelerated for the I-group, whereas the rates were similar between the groups until that time point. After 36 months, around 22% of patients in the C-group had not RTW, whilst this number was around 10% for the I-group.

### Comparison with Previous Studies

The results of our study align with the results from the original RCT with a wait-list control that took place in Aarhus, Denmark in 2006–2009 [20]. This RCT showed a mean reduction of 7 weeks of sick leave for the intervention group in weeks 1–48 and a faster RTW rate (HRR 1.58 (95% CI: 0.89–2.81)) for the intervention group. However, the results did not reach statistical significance [20]. One plausible reason for this difference between studies may be sample size, as Willert et al. recruited 51 participants pr. group, we included 221 patients in the C-group and 331 patients in the I-group. Willert et al. observed a mean reduction of three sick leave weeks during the first 16 weeks [20]. In contrast, we did not find any difference in sick leave weeks between groups during the first 6 months. This delayed effect of the intervention we observed may be because the I-group waited up to 6 months to start the intervention after their assessment at DOEM, which may have postponed RTW. If we change the start date for our analyses to the actual start date for the intervention, the HRR increases to 1.41 (1.15–1.73).

In a meta-analysis of CBT-based interventions and their effect on RTW for people on sick leave, Xu et al. found that CBT reduced the length of sick leave and increased the number of people who RTW [13]. This is in line with our findings. Xu et al. also observed a reduction in stress symptoms for the intervention group and argued that the decrease in sick leave may be due to improved mental health and thus, more energy and willingness to RTW.

### Strength and Weaknesses

A key strength of our study is the utilization of register-based data from the DREAM register, which provides an objective source of information not prone to loss to follow-up or recall bias. However, a limitation is that the DREAM register only includes long-term sick leave, as the first 30 days of sick leave benefits are covered by the workplace. As a result, our findings may underestimate the total duration of sick leave, although this underestimation is consistent across all groups. Another strength is the clearly defined study population which was assessed by trained psychologists. A retrospectively selected number of patients were used for the C-group in the study which is a potential weakness in the design. Patients who would have been eligible to receive the intervention were selected for the C-group based on a screening of patient journals from 2011 to 2012 by one of the psychologists at DOEM. However, it is not every eligible patient who accepts the offer of the intervention for various reasons, e.g. negative attitudes towards a group-based intervention. The C-group may thus include a broader range of patients with different attitudes than the I-group. This may introduce a positive bias in the interpretation of our results as the C-group may have been less motivated and susceptible to the intervention than the I-group. We cannot account for whether this may affect the outcomes; however, the baseline characteristics of the two groups were comparable and the characteristics that differed between the groups did not result in a changed HRR following the adjusted analysis. Other factors such as contextual or organizational factors within the workplace may also affect the intervention outcome; however, as such variables were not available in the dataset, we were unable to investigate any mediating effect. Further, the participant characteristics resemble the patient characteristics described in the study by Willert et al. [20]. The overrepresentation of women in this study should be considered when extrapolating the findings to the broader population.

Another weakness in our design is that the C-group remains fixed in time in 2011–2012, whilst the I-group spans 2011–2018. In 2013, a new Danish law reduced the maximum number of weeks on sick leave with full economic compensation from 52 to 22 weeks. However, in our second sensitivity analysis, we found that including only patients from before 2014 or from 2014 to 2018 in the I-group did not affect the hazard ratio.

The DREAM-database contains various types of public benefits with various implications for our analyses of RTW. In our analysis, we categorized a negative outcome as any type of public benefit except educational grants, retirement, emigration, death, or maternity/paternal leave. Consequently, those who end up working in a position such as the flexjob, which is a partly subsidized job tailored to workers with reduced capacity for work, is counted as a negative outcome. The flexjob may, however, be a positive outcome for those patients who would otherwise depend on public benefits alone. Our analysis is thus a conservative estimate of the potential benefits of receiving the intervention.

Our study has demonstrated a significant effect of the CBT intervention to significantly reduce cumulated sick leave weeks and facilitate faster RTW. More research to understand how and under what circumstances the intervention produces the effects observed in this study will warrant further investigation.

## Supplementary Information

Below is the link to the electronic supplementary material.

Supplementary file 1 (DOCX 372 KB)

## Data Availability

The data used in this study was obtained from national registers which are not publicly available due to privacy and confidentiality restrictions. Access to the data may be granted upon request through Statistics Denmark. Additionally, access to the Danish Occupational Medicine Cohort can be granted upon reasonable request by contacting the Department of Occupational Medicine at Aarhus University Hospital, Denmark.
