# Supplementary material for: Effect of Cognitive Behavioural Stress Management on Return-to-Work Amongst Sick-Listed Employees
Source: J Occup Rehabil. 2025 Jun 25;36(3):729–36. doi: 10.1007/s10926-025-10306-2 (PMC13364782; doi:10.1007/s10926-025-10306-2)
Supplement: Supplementary file 1 — (DOCX 372 KB) [file 10926_2025_10306_MOESM1_ESM.docx]

Supplementary file: The stress management intervention (source: authors’ own work (1))

1. Bond CB, Brandt LPA, Ditlevsen DN, Dalkin S, Willert MV, Andersen LN. Realist evaluation of a CBT-based stress management intervention for Danish patients on sick leave due to work-related stress: A study protocol. Evaluation and Program Planning. 2025;112:102634.
